# A Case of Fatal Phlegmasia Cerulea Dolens: Challenges in Its Management

**DOI:** 10.7759/cureus.91833

**Published:** 2025-09-08

**Authors:** Alex S Nguyen, Peter W Deucher, Jamie Hill, Kotikalapudi Sivarama

**Affiliations:** 1 Pathology, William Carey University College of Osteopathic Medicine, Hattiesburg, USA; 2 Medicine, William Carey University College of Osteopathic Medicine, Hattiesburg, USA; 3 Internal Medicine, Merit Health Wesley Hospital, Hattiesburg, USA

**Keywords:** compartment syndrome, deep vein thrombosis, endovenous thrombectomy, phlegmasia cerulea dolens, reperfusion injury

## Abstract

Phlegmasia cerulea dolens (PCD) is a rare condition that poses significant challenges due to its high mortality and morbidity rate and lack of formal, well-developed treatment protocols. PCD presents as a massive deep vein thrombosis associated with compartment syndrome of the lower extremities. This case report outlines the clinical course of a 64-year-old morbidly obese female patient with a history of hypertension, dyslipidemia, hypothyroidism, and pulmonary embolism who presented to the emergency department with PCD. Despite thrombolytic therapy, oral anticoagulation, and endovenous thrombectomy, the patient’s condition only worsened, resulting in refractory shock and death. This case highlights the significance of recognizing PCD early, anticipating its complications, and refining management strategies to improve patient outcomes.

## Introduction

Phlegmasia cerulea dolens (PCD) is a rare and challenging clinical scenario characterized by massive venous thrombosis and near-total venous occlusion of the extremities [1]. PCD carries a high incidence of pulmonary embolism (PE), leg amputation, and mortality [1]. PE occurs in up to 50% of these patients with PCD [2], while the risk of leg amputation with PCD is between 10 and 25% [2] but can be as high as 50% if there is coexisting venous gangrene [3]. 

PCD typically affects the left lower limb, which is four times more likely to be involved compared to the right [1,4]. This is attributed to the compression of the left iliac vein by the right iliac artery, leading to an anatomical predisposition for venous stasis in the left lower extremity [1,5]. Major risk factors for PCD include cancer, hypercoagulable states, a history of deep vein thrombosis (DVT) or PE, trauma, advanced age, obesity, and prolonged immobility [5]. Additionally, PCD occurs more in men than in women [5]. 

Gregorie first described the term "phelgmasia cerulea dolens" in 1938, which translates to “painful blue inflammation” [1,5,6]. On the same spectrum of disease is phlegmasia alba dolens, which is milder and does not affect collateral circulation. PCD is split up into three stages: venous stasis with congestion, arterial repercussions, and extensive gangrene [4]. The diagnosis of PCD is primarily established by clinical examination, which reveals several hallmark factors such as skin edema, pain out of proportion, cyanosis, or skin mottling [5]. 

## Case presentation

Day 1 

A 64-year-old woman presented to the emergency department via ambulance with pain and swelling of the left leg over the last three months that intensified early that morning when she woke up. The patient had a medical history of PE, essential hypertension, hypothyroidism, and dyslipidemia. In addition to leg pain, she reported reduced sensation and mobility in her left lower extremity. She had been given 10 mg of IV morphine en route to the hospital. Of note, the patient reported that she was treated with thrombolytic therapy followed by oral anticoagulation for her previous PE. The oral anticoagulation was stopped after six months. She denied any history of thrombophilia. The patient reports that, over one year on occasion, while sitting at the kitchen table, she has experienced numbness to the left lower extremity. 

**Figure 1:**
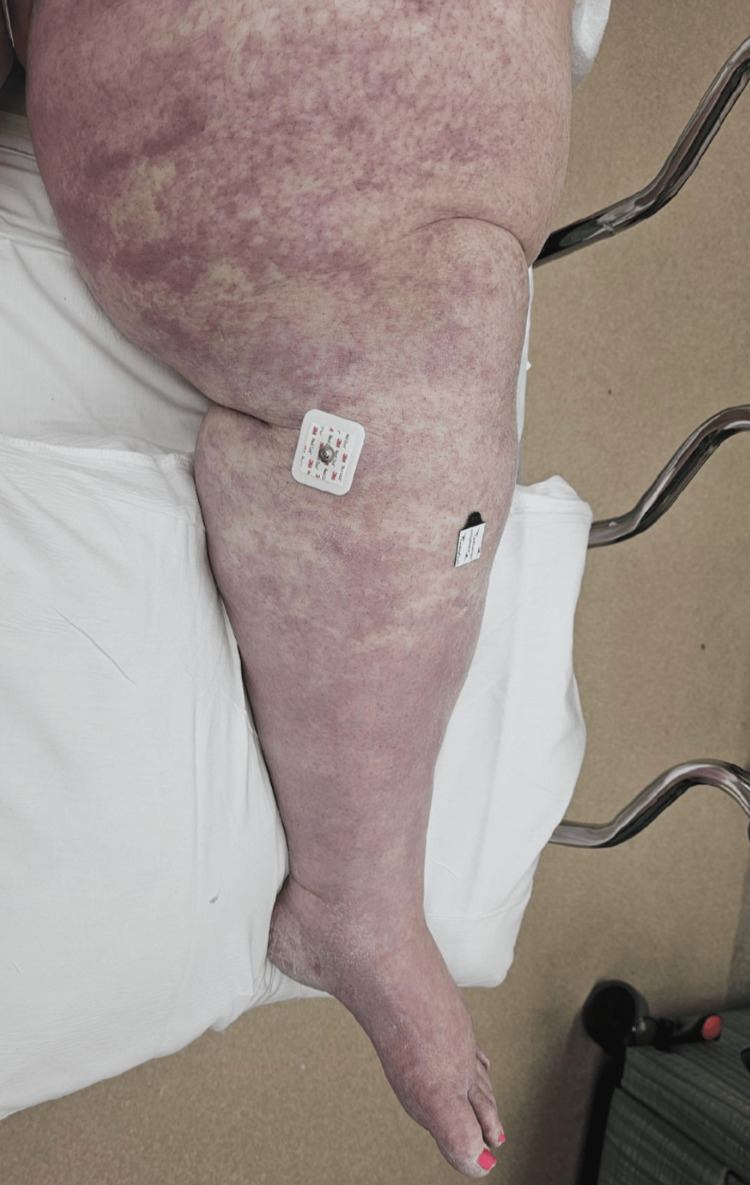
Patient’s Left Leg on Presentation

**Figure 2:**
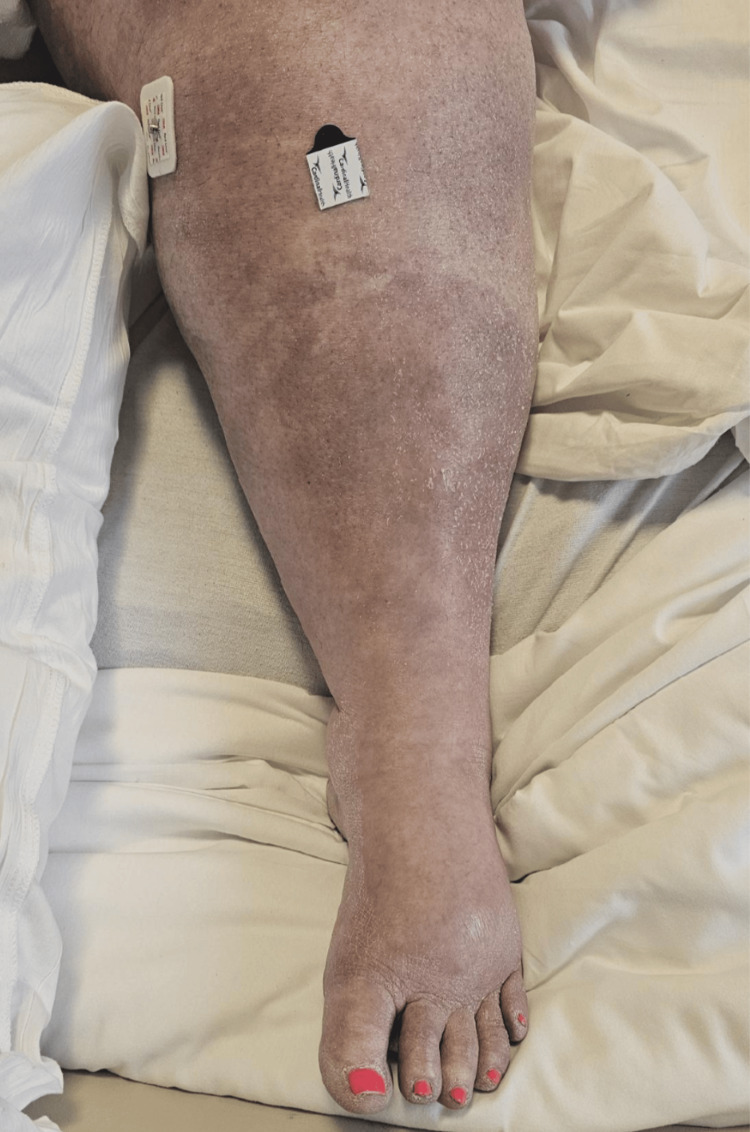
Patient’s Left Leg on Presentation

On arrival, her vital signs were as follows: blood pressure of 94/45 mmHg, heart rate of 110 beats per minute, respiratory rate of 19 breaths per minute, and temperature of 98 °F. A second blood pressure reading only minutes later was 132/62 mmHg. A physical exam revealed a massively swollen, tender, cold, and cyanotic left leg (Figures 1, 2). She demonstrated decreased sensation and mobility in her toes. A posterior tibial pulse was only faintly present with Doppler. 

**Figure 3:**
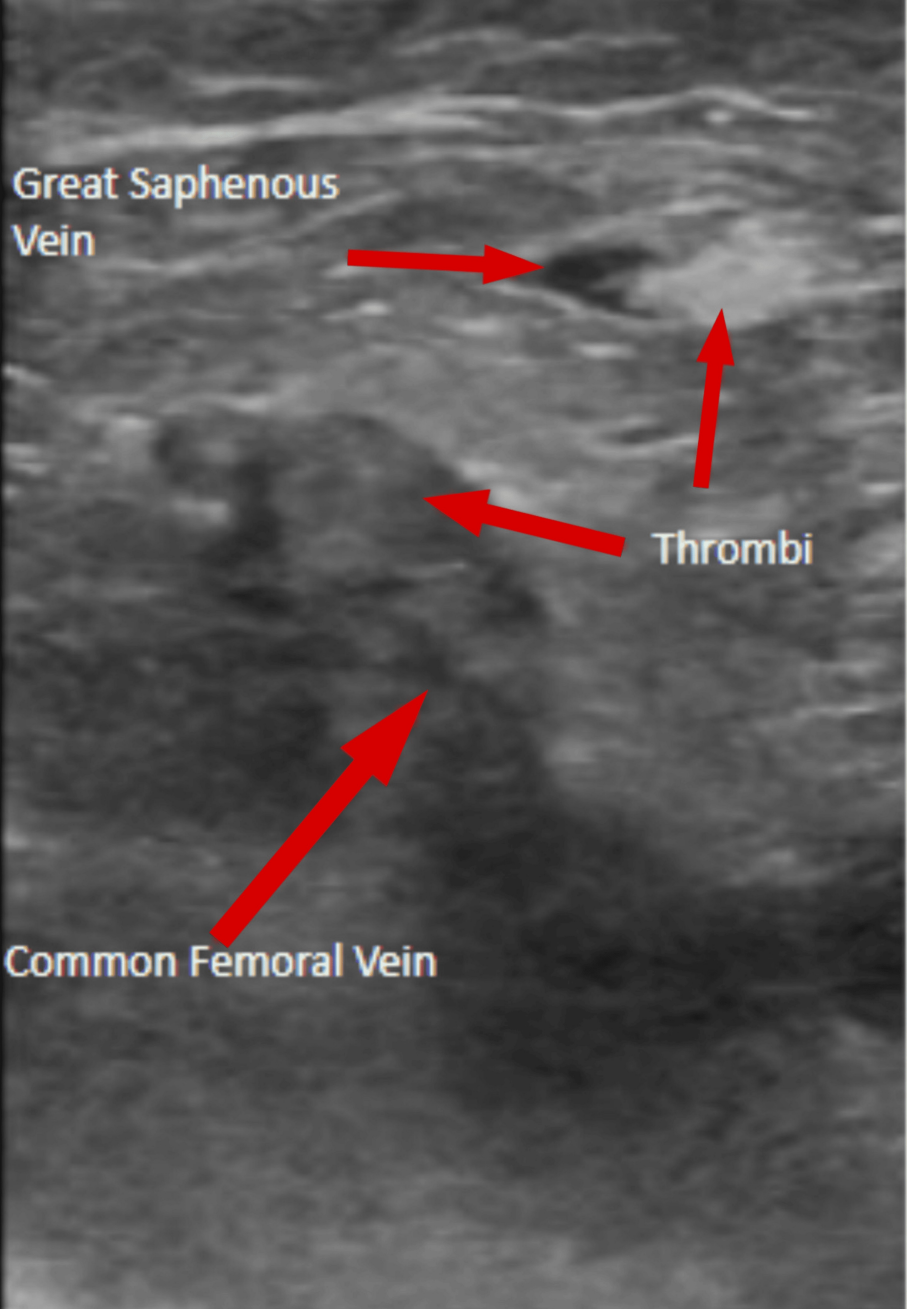
Ultrasound of the Left Lower Extremity Showing Thrombi

Initial lab findings reveal leukocytosis with neutrophilia, elevated D-dimer, hyperkalemia, elevated creatinine, hypomagnesemia, and elevated lactic acid (Table 1). A lower left extremity venous ultrasound in the ED revealed an acute occlusive DVT affecting the common femoral, popliteal, anterior tibial, and peroneal veins, as well as occlusive superficial thrombophlebitis of the great saphenous vein (Figure 3). Chest X-ray showed calcification of the thoracic aorta. The patient was treated with lactated Ringer's bolus (1,000 mL IV) and empiric ceftriaxone (2,000 mg IV) once to cover for infection. For pain management, fentanyl (50 mcg IV) and hydromorphone (2 mg IV) were administered. To treat the left leg DVT, heparin IV was started, and cardiology was consulted. 

**Table 1:** Patient's Laboratory Values on Arrival to the ER Source: [7]

Laboratory Parameters	Patient Values	Reference Ranges
White Blood Count	20.10	4000 to 11,000/microL
Red Blood Count	3.84 x 10^6/mcl	4.2 to 5.9 million red blood cells/microL
Hemoglobin	10.5	Female: 12 to 16 g/dL
Hematocrit	35.2	Female: 37 to 47 percent
Platelet	338	150,000 to 450,000/microL
Neutrophils% Auto	88.4 %	50 to 70 percent
Neutrophils # Auto	17.8 x 10^3/mcL	2000 to 8250/microL
Lymphocytes % Auto	5.9 %	30 to 45 percent
Sodium	135	136 to 145 mEq/L
Potassium	4.6	3.5 to 5 mEq/L
Carbon Dioxide	17	23 to 30 mEq/L
Chloride	104	98 to 106 mEq/L
Creatinine	1.42	Female: 0.50 to 1.10 mg/dL
Blood Urea Nitrogen	15	8 to 20 mg/dL
Glucose	241	70 to 99 mg/dL
Magnesium	1.5	1.6 to 2.6 mg/dL
Calcium	9	8.6 to 10.2 mg/dL
Prothrombin Time	12	17 to 23 seconds
International Normalized Ratio	0.99	0.8 to 1.2
Partial Thromboplastin Time	27.20	25 to 35 seconds

The patient was admitted from the ED to the telemetry unit for close monitoring. After admission, the patient was taken to the catheterization lab, where a support catheter was placed in the popliteal vein to infuse alteplase. However, an ultrasound-assisted thrombolysis catheter could not be advanced using a stiff guidewire. The decision was made to restudy in the catheterization lab the next day. 

Day 2 

In the catheterization lab later that evening, a venogram was performed followed by a thrombectomy and a successful placement of an iliac stent. The patient was found to have severe external iliac stenosis. Heparin was discontinued and apixaban was initiated. The patient experienced low blood pressure and significant blood loss, prompting consideration of a possible blood transfusion. Her hemoglobin was 9.6 g/dL. The right groin was unremarkable, with no signs of hematoma. To evaluate the patient’s chest pain, a chest X-ray was performed, which revealed subtle increases in density in the lung bases.

Day 3 

The patient experienced increased restlessness the following morning. A central line was placed in her right internal jugular vein due to her worsening hypotension and lactic acidosis. Soon after, the patient was intubated due to acute hypoxic respiratory failure. A retroperitoneal hematoma was suspected as a possible complication of her iliac stent placement, but CT could not be obtained to confirm due to her obesity. Her hemoglobin dropped to 7.7 g/dL that afternoon, but blood was not transfused. Her pressors were increased. The patient had been made DNR and later in the day, asystole ensued, and the patient died. 

## Discussion

PCD is a rare but life-threatening condition characterized by massive venous thrombosis, obstructed outflow, leading to arterial collapse, and in severe cases, gangrene. There is no standardized treatment protocol for PCD, which contributes to the high rates of serious complications. These complications occur due to extensive venous obstruction followed by tissue ischemia. Reported complications include venous gangrene in 40% - 60%, PE in 50%, and amputation in 10%-25% of patients with this condition [2]. The mortality rate increases to 20%-40% and the risk of amputation is 20%-50% with venous gangrene [3]. The present case highlights the need for prompt diagnosis and treatment, as the patient did not seek medical attention until roughly 24 hours after the onset of symptoms. 

The patient presented with many known PCD risk factors, such as prior PE, suspected thrombophilia, obesity, and advanced age. While malignancy is one of the main predisposing factors, it was not ruled out in this case. The patient’s rapid deterioration illustrates a crucial gap in current treatment protocols. 

A notable contributor to the patient’s decline may have been reperfusion syndrome, which previous studies have recognized as a complication following revascularization in PCD [8-10]. Reperfusion syndrome is marked by tissue damage, inflammation, and oxidative stress that occurs when blood flow returns to previously ischemic tissues [9]. Lab findings that point to a reperfusion injury in this patient include a white blood cell count of 40.30x103/mcl with neutrophil predominance of 97.3%, a decreased but still within-range platelet count, which dropped from 338 × 10³/µL to 158 × 10³/µL, and elevated urea and creatinine levels, which were recorded at 43 mg/dL and 4.74 mg/dL, respectively. Furthermore, lactic acid levels rose significantly on day 3, reaching a level of 11.5 mmol/L, compared to a normal level of 1.6 mmol/L on day 2. This notable increase in lactate points to tissue hypoxia and anaerobic metabolism, a sign of reperfusion injury. 

Earlier catheter-directed thrombolysis or preemptive surgical interventions have been linked with better outcomes in other cases of PCD [1,11]. Some patients may undergo aggressive treatments such as fasciotomy or amputation yet in our case amputation was never discussed, most likely due to the limb appearing viable. This poses a question of whether limb viability alone can establish management or should systemic signs such as worsening shock prompt consideration of earlier amputation. This case highlights a critical gap in the management of PCD, most specifically, the need for more explicit guidance regarding when to escalate care in the setting of systemic deterioration without gangrene.

## Conclusions

This case illustrates a fatal course of PCD in a 64-year-old woman with multiple comorbidities and serves to highlight the need for improving and formalizing management protocols. This case serves as a reminder of the careful consideration necessary for early detection and intervention to prevent DVTs from progressing to PCD. Current medical management for PCD includes initiation of anticoagulation with unfractionated heparin and supportive care for pain. When anticoagulation fails, endovascular and surgical interventions such as catheter-directed thrombolysis or percutaneous mechanical thrombectomy are considered. Furthermore, the case exemplifies how endovascular thrombectomy can potentially contribute to morbidity and mortality via reperfusion injury or excessive bleeding. Finally, this case serves as a reminder that determining when to escalate from medical and endovascular management to limb amputation is a difficult but crucial process. Further research is needed to clarify the optimal timing and type of management for this serious, albeit rare condition. 
